# Arabidopsis brassinosteroid biosynthetic mutant *dwarf7-1 *exhibits slower rates of cell division and shoot induction

**DOI:** 10.1186/1471-2229-10-270

**Published:** 2010-12-09

**Authors:** Jinyeong Cheon, So-Young Park, Burkhard Schulz, Sunghwa Choe

**Affiliations:** 1 School of Biological Sciences, College of Natural Sciences, Seoul National University, Seoul 151-747, Korea; 2 Biotechnology Division, Korea Forest Research Institute, Kwonseon-Gu, Gyeonggi-Do, Suwon 441-350, Korea; 3 Department of Horticulture and Landscape Architecture, Purdue University, West Lafayette, IN 47907, USA; 4 Plant Genomics and Breeding Institute, Seoul National University, Seoul 151-921, Korea

## Abstract

**Background:**

Plant growth depends on both cell division and cell expansion. Plant hormones, including brassinosteroids (BRs), are central to the control of these two cellular processes. Despite clear evidence that BRs regulate cell elongation, their roles in cell division have remained elusive.

**Results:**

Here, we report results emphasizing the importance of BRs in cell division. An Arabidopsis BR biosynthetic mutant, *dwarf7-1*, displayed various characteristics attributable to slower cell division rates. We found that the *DWARF4 *gene which encodes for an enzyme catalyzing a rate-determining step in the BR biosynthetic pathways, is highly expressed in the actively dividing callus, suggesting that BR biosynthesis is necessary for dividing cells. Furthermore, *dwf7-1 *showed noticeably slower rates of callus growth and shoot induction relative to wild-type control. Flow cytometric analyses of the nuclei derived from either calli or intact roots revealed that the cell division index, which was represented as the ratio of cells at the G2/M vs. G1 phases, was smaller in *dwf7-1 *plants. Finally, we found that the expression levels of the genes involved in cell division and shoot induction, such as *PROLIFERATING CELL NUCLEAR ANTIGEN2 *(*PCNA2*) and *ENHANCER OF SHOOT REGENERATION2 *(*ESR2*), were also lower in *dwf7-1 *as compared with wild type.

**Conclusions:**

Taken together, results of callus induction, shoot regeneration, flow cytometry, and semi-quantitative RT-PCR analysis suggest that BRs play important roles in both cell division and cell differentiation in Arabidopsis.

## Background

Plant steroidal hormones, brassinosteroids (BRs), are central to supporting the proper growth and development of plants. As a result, BR biosynthetic and response mutants exhibit phenotypes characterized by severe growth deficiencies. Mutants of various species, including Arabidopsis, pea, tomato, rice, barley, and morning glory, have been found and shown to display similar phenotypes of growth deficiency [1-5].

Brassinolide (BL), the most active BR and an end product of the BR biosynthetic pathway in Arabidopsis, is synthesized from sterols, including campesterol or cholesterol [6]. Of the enzymes involved in BR biosynthesis, the C22-α-hydroxylase DWARF4 (DWF4) mediates a rate-determining step [7,8]. After going through this step, intermediates possess dramatically increased bioactivities [6]. As such, the enzymatic steps could be classified as enzymes active before and after DWF4. The enzymes DWARF1/DIM1/CBB1 [9], DWARF5 [10], DWARF7 [11], and DE-ETIOLATED2 [12-14] act before DWF4, whereas CONSTITUTIVE PHOTOMORPHOGENESIS AND DWARFISM (CPD) [15,16], ROTUNDAFOLIA3 (ROT3) [17,18], Cytochrome P450 (CYP90D1) [19] and BR6-oxidase (BR6Ox) [20-28] are active after DWF4. Depending on the species and especially in rice, BR biosynthetic pathways culminate at castasterone (CS) which serves as the primary bioactive BR, rather than BL [20]. The two bioactive BRs in Arabidopsis, CS and BL, are perceived by a plasma membrane-localized receptor complex composed of BRI1 and BAK1 [29-32]. Upon phosphorylation and activation by BRs, the receptor complex dissociates a negative regulator BRI1 KINASE INHIBITOR1 (BKI1) [33]. BRI1 SUPPRESSOR1 (BSU1), which is a protein phosphatase with a Kelch-repeat domain, is bound by activated BSK1 [5,34] to deactivate the negative regulator BRASSINOSTEROID-INSENSITIVE2 (BIN2) [35-38], diminishing its negative regulatory effects [34].

The transcription of BR-dependent genes is regulated by a plant-specific family of transcription factors including BRASSINAZOL-RESISTANT1 (BZR1) [39] and BRI1-EMS-SUPPRESSOR1 (BES1) [40,41] in Arabidopsis. Although BES1 and BZR1 share 88% identity at their amino acid sequences, the two transcription factors regulate their target genes differently; BES1 is involved in transcriptional activation [40], and BZR1 both activates and represses transcription [39,42]. As such, constitutive BR phenotypes are seen in the *bes1-D *mutant [40], whereas the semi-dwarf phenotype is a characteristic of the light-grown *bzr1-D *mutant due to the repression of its target gene, *DWF4 *[42].

As compared with the roles that BRs play in cell elongation, their effects on cell division have not received as much focus in studies to date. Earlier research suggested that BRs stimulate cell division [43-46], which was based on observations of the effects of BRs on cultures of suspension cells or protoplasts. At the molecular level, it was found that the stimulation of cell division in the BR biosynthetic mutant *de-etiolated2 *results from the activation of the *CycD3 *gene in Arabidopsis [47]. In addition to the callus or protoplast system, clearer evidence was provided by a recent paper showing that BR-deficient mutants exhibit fewer numbers of cells in the provascular ring of inflorescences, resulting in a reduced number of vascular bundles in these mutants [48].

Using Arabidopsis mutants that are defective in BR biosynthesis, *dwf7-1*, we investigated the role of BRs in cell division. We examined the differences in the establishment of mutant-derived calli, shoot regeneration from calli or directly from root explants. In addition, we employed flow cytometric analyses to look at cell cycle progression. Finally, the transcript levels of the genes involved in cell division and cell differentiation were tested in wild type and BR mutants. Our results provide evidence that BRs actively regulate cell division in Arabidopsis.

## Results and Discussion

### A BR biosynthetic mutant displays differences in callus induction rate

**Figure 1:**
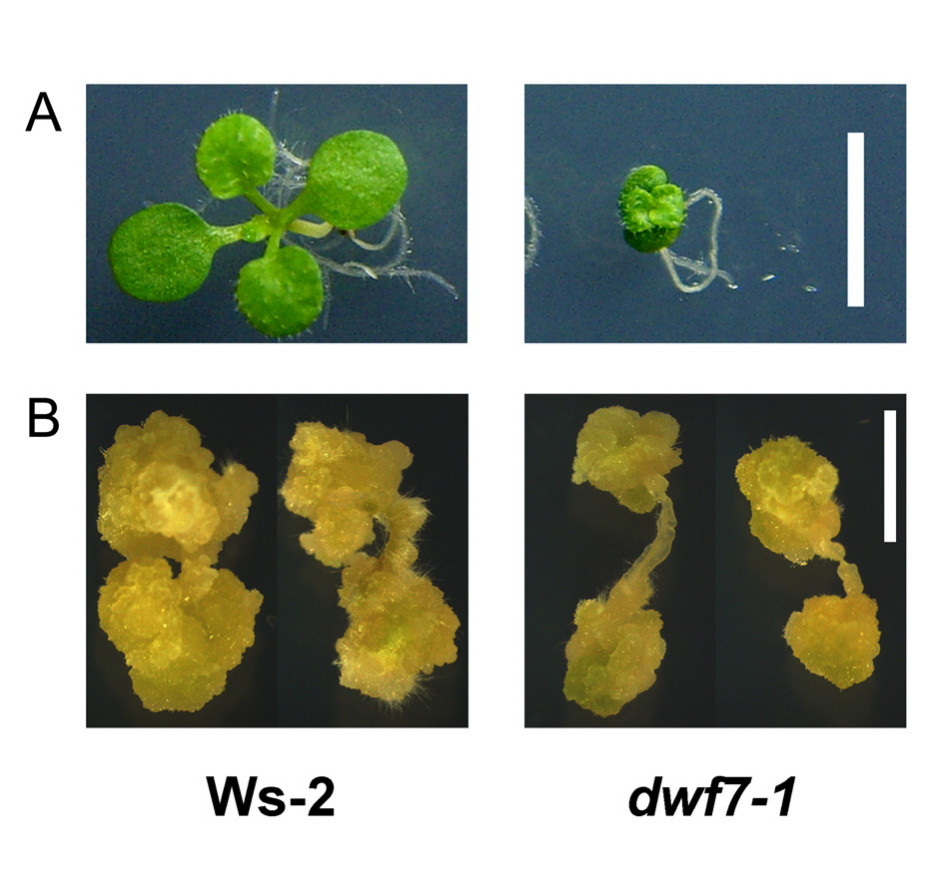
**Morphology of seedlings and calli induced from each genotype**. (A) Phenotypes of Ws-2 wild type and the *dwf7-1 *mutant. As compared with wild type, *dwf7-1 *exhibited severe dwarfism. Scale bar = 0.5 cm. (B) Callus morphology resulting from the different genotypes. Each genotype successfully established calli when the explants were grown for 30 days in callus induction medium. The sizes of *dwf7-1 *calli were relatively small compared to the wild-type calli. Unit bar = 0.2 cm.

The exogenous application of brassinosteroids was previously shown to simulate cell division during callus culture. To test whether the endogenous alteration of the BR levels affects the callus induction and cell division rates, a mutant defective in BR biosynthesis, *dwf7-1*, was subjected to callus induction. Figure 1A illustrates the seedling phenotypes of the Ws-2 wild type and *dwf7-1*. As compared with the wild type, *dwf7-1 *exhibited more severe dwarfism due to defects in BR biosynthesis. When calli were induced from root explants, BR mutants successfully established visually discernable calli after 15 days. Figure 1B displays the morphology of the representative calli grown for 30 days after the induction from root explants. Noticeably, the size of calli from *dwf7-1 *is smaller relative to the wild type (Figure 1B).

The slower growth of the mutant could be due to either slower cell division or slower cell growth or both, but in any case, it is obvious that BRs are important to this process.

### The BR biosynthetic gene *DWARF4 *is actively expressed in calli

To examine whether BRs are required during callus establishment, we checked the expression level of the *DWF4 *promoter-GUS reporter gene [49]. As part of an important rate-determining step, *DWF4 *expression was proposed to represent tissues with enriched BR levels [49].

**Figure 2:**
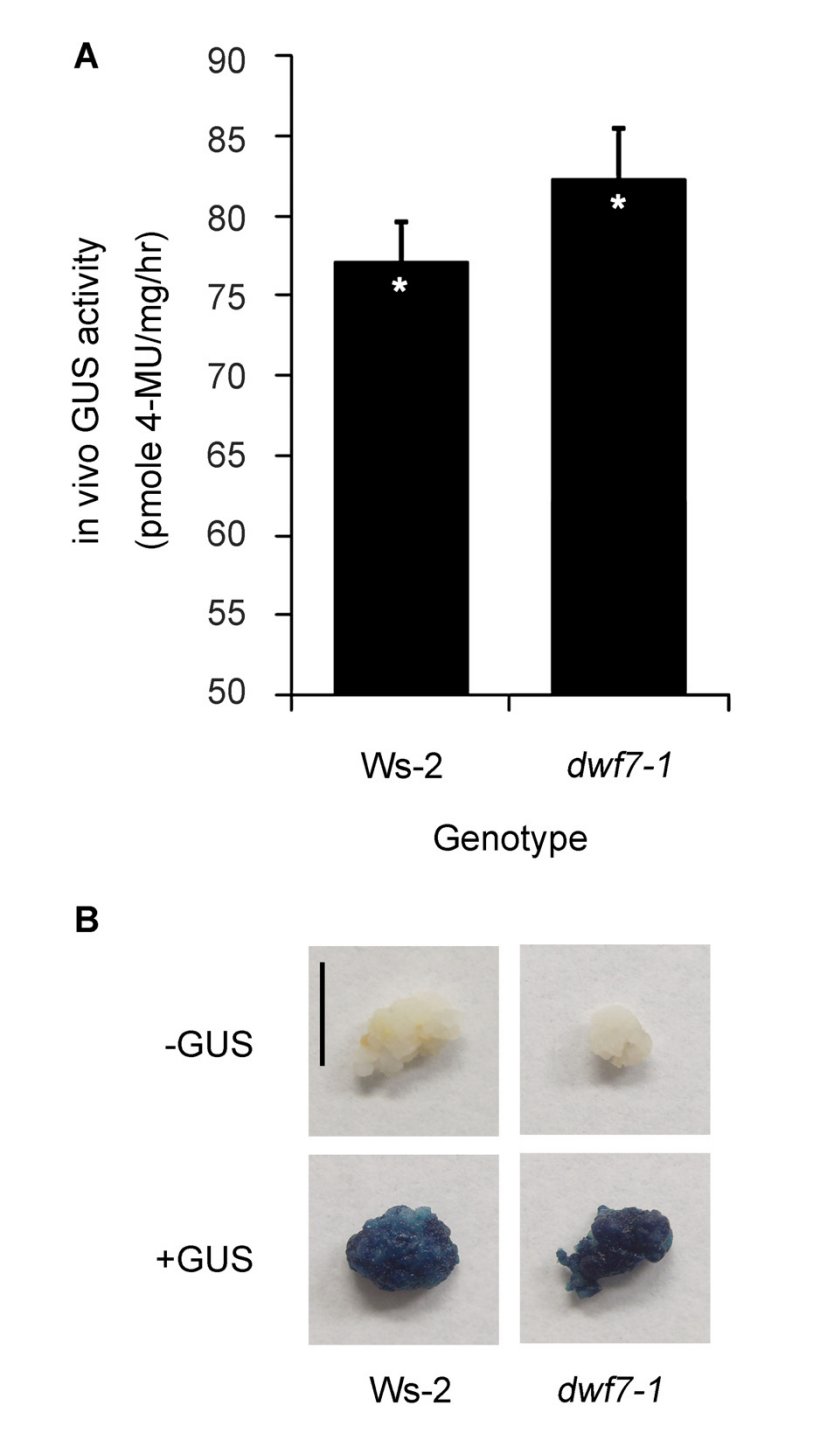
**Different levels of *DWF4pro:GUS *expression depending on genotypes**. (A) In vivo GUS assay of *DWF4pro:GUS *activity in different genetic backgrounds. As compared with the wild type, its activity was slightly increased in *dwf7-1*. However, the responses of Ws-2 and *dwf7-1 *were not significantly different, as denoted by one asterisk (Student's t-test, n > 10, p < 0.05). (B) GUS histochemical staining pattern of *DWF4pro:GUS *for each genotype. Consistent with the results in panel A, the blue staining was more evident in *dwf7-1*. Calli shown are from wild type and *dwf7-1*, from left. Top row displays the control calli that were not stained with GUS. Bottom row shows calli after GUS histochemical staining. Unit bar = 1 cm.

First, in vivo GUS assays revealed that *DWF4 *is strongly expressed in both wild-type and *dwf7-1 *calli [42]. This in vivo GUS assay was further visualized by GUS histochemical staining (Figure 2B). The dark blue staining, signifying GUS activity, was prominent in both the wild type and *dwf7-1 *relative to the control calli that were not stained with GUS (Figure 2B). Because we previously found that exogenously supplied auxin resulted in *DWF4 *induction, we examined another set of calli after washing off the auxin that had been added to the callus induction medium (CIM).The washed calli also displayed a similar pattern (additional file 1: GUS staining pattern after auxin washing). Both the results from the GUS histochemical assay and the in vivo GUS activity tests imply that BRs are required to maintain the calli status [49].

### The ratios of cells at G2/M vs. G1 phases are low in *dwf7-1*

**Figure 3:**
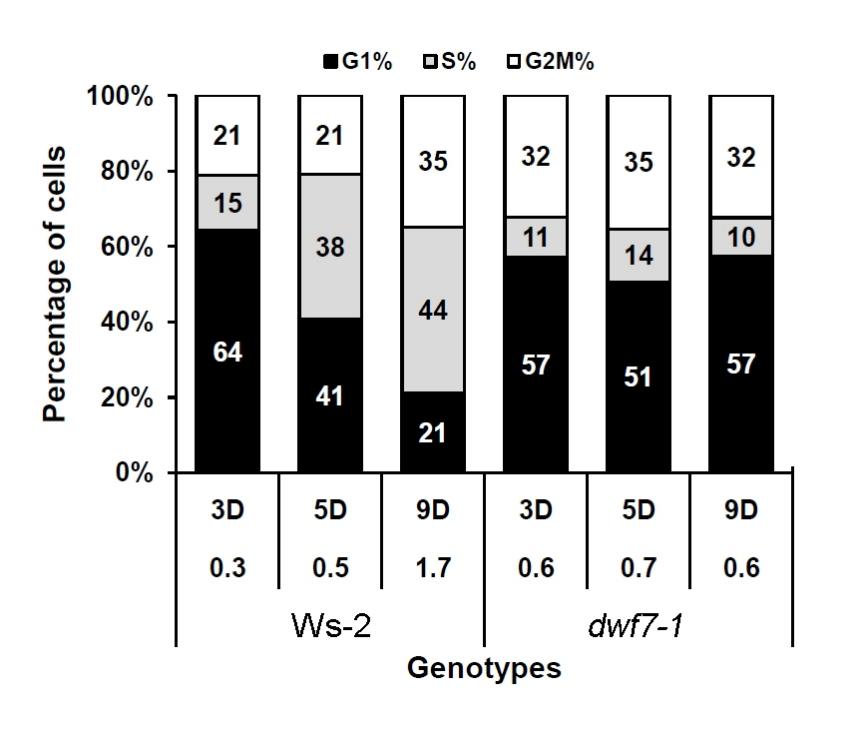
**Flow cytometry-based time-course analysis of the DNA profile in the nuclei derived from different genotypes of calli**. Nuclei isolated from calli grown for 3, 5, and 9 days after transferring to fresh media were subjected to flow cytometry. Percentage of DNA profile corresponding to G1, S, and G2/M phases are plotted in the stacked column chart. Numbers in the stacked column are percentage of each phase. The numbers below each column indicate G2/M vs. G1 ratios. Shown are triplicate experiments of at least 5,000 nuclei.

Because we observed that BR mutants display different rates of callus induction, we tested whether this is associated with a difference in cell division rates. To do this, we measured the DNA content in the nuclei of each callus using flow cytometry. Figure 3 illustrates the results with a 100% stacked column chart. The percentage of cells in the three phases of the cell cycle--G1, S, and G2/M--were evaluated by the Partec software.

When cell division indices (ratio of G2M% vs. G1%) were compared, the portion of wild-type cells in G1 phase gradually decreased with time, whereas the portion in S and G2/M phases increased. This suggests that wild-type cells are synthesizing DNA and dividing until the 9th day after transfer to fresh medium. In *dwf7-1 *cells, both phases remained relatively stable; G2/M at their 30s and G1 at 50s percentage.

The ratios of G2/M vs. G1 were then compared. Wild type increased from 0.3 at day 3 to 1.7 at day 9. However, the ratios were relatively stable in *dwf7-1 *cells, holding at 0.6-0.7 regardless of time, suggesting that it takes longer for *dwf7-1 *callus cells to finish one round of cell division.

### A BR mutant displays differential level of shoot induction from calli

We next aimed to test whether a BR deficiency affects the shoot induction rate. Two different routes were taken; shoots were induced from calli and directly from root explants.

**Figure 4:**
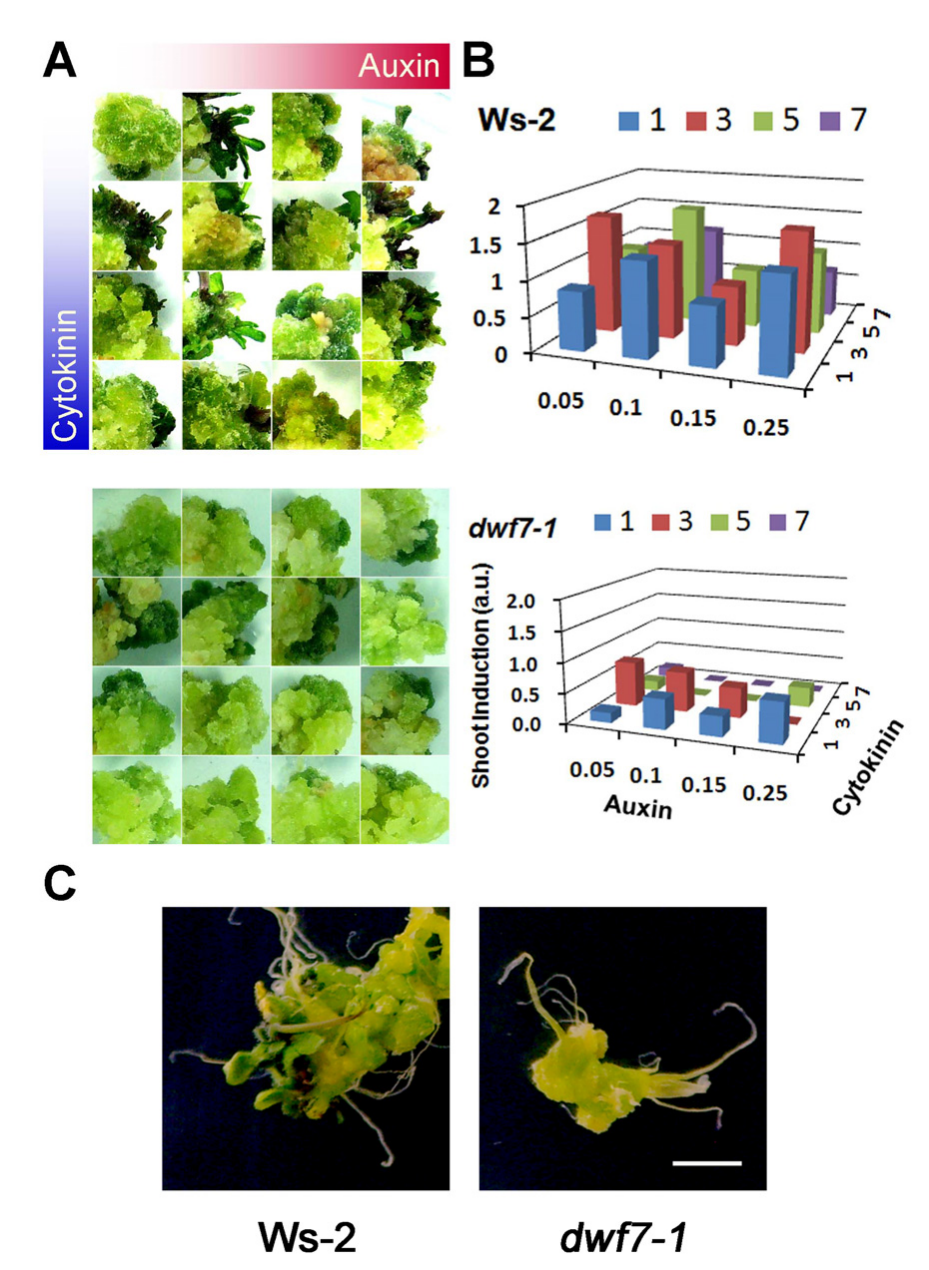
**Organogenesis patterns on a matrix of shoot induction medium**. (A) Representative calli grown on each combinatorial medium. The 16 different combinations of IAA (0.05, 0.1, 0.15, and 0.25 mg/L) and N^6^-Δ^2^-isopentenyladenine (1, 3, 5, and 7 mg/L) were tested with each callus. 3-9 calli per each concentration were tested. Shown are composite photos of calli arranged after growth on each medium. (B) Degree of shoot induction in specific medium. The labeling for the three axes shown on the *dwf7-1 *chart is applicable to the Ws-2 graph. Arbitrary unit representing shoot induction rate, ranging from 0 to 2, was plotted against the combination of auxin and cytokinin. A number close to 2 indicates that each callus tested on the specific medium tended to regenerate shoots successfully. (C) Direct induction of shoots from root explants. Roots were briefly exposed to callus induction media, then transferred to shoot induction media to induce shoots from each genotype. Wild type displays discernable structures of leaves and inflorescences, whereas these morphologies are relatively primordial in *dwf7-1 *mutant. Scale bar = 0.5 cm.

First, the calli established from each genotype were subject to shoot regeneration in shoot induction medium (SIM) with different combinations of auxin and cytokinin concentrations; auxin concentrations of 0.05, 0.1, 0.15, and 0.25 mg/L and cytokinin concentrations of 1, 3, 5, and 7 mg/L. On each medium of the 16 combinatorial SIMs, three to nine calli were grown for 4 weeks to establish shoots. One representative was chosen for illustration in the Figure 4A. Overall, wild-type calli produced shoots at broad ranges of auxin and cytokinin concentrations. As visible evidence of shoot induction, the calli turned green and produced elongated inflorescences with leaf structures. *dwf7-1 c*alli displayed only marginal signs of shoot induction; parts of calli turned green, but almost no shoots were produced (Figure 4A).

To present the results more quantitatively, we transformed the degree of shoot induction into arbitrary numbers ranging from 0 to 2 and displayed them using three dimensional charts (Figure 4B). We assigned the numbers 0, 1, and 2 to calli having no shoots, greening only, and visible shoots, respectively; thus, a number close to 2 meant that most of the calli tested at the specific combination of auxin and cytokinin produced visible shoots, whereas a number closer to 0 indicated that none of the calli formed visible shoots. Of the 16 different concentration combinations, the wild type produced shoots at the broadest ranges (Figure 4B, Ws-2). However, shoot induction was noticeably low in *dwf7-1 *calli.

Shoots were also directly induced from root explants. Figure 4C summarizes the results. Wild type displayed vigorous shoot induction; explants produced visibly elongated inflorescences with leaves. In contrast, *dwf7-1 *calli barely displayed shoot induction; developed calli turned green but made only primordial leaves. The lower rates of shoot induction observed in the mutant suggests that BRs are central to controlling cell differentiation as well as division.

### Populations of dividing cells are smaller in the roots of *dwf7-1 *relative to wild-type cells

**Figure 5:**
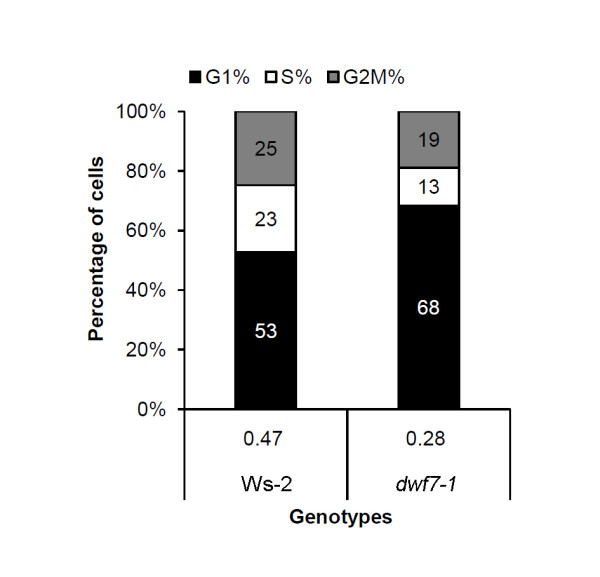
**DNA profile in the root cells of *dwf7-1 *and its parental wild type**. Flow cytometry-based DNA profiles in the nuclei derived from roots of different genotypes were determined. Percentage of DNA profile corresponding to G1, S, and G2/M phases are plotted in the stacked column chart. Numbers in the stacked column are percentage of cells in each phase. The numbers below each column indicate ratios of G2/M vs. G1. Shown are triplicates of at least 3,000 nuclei.

To examine cell division in the roots of intact plants, the percentage of cells in the three phases of the cell cycle--G1, S, and G2/M--were evaluated with the Partec software. The results are summarized in Figure 5. When Ws-2 wild-type and *dwf7-1 *cells were compared, the ratios of cells in the three phases clearly showed differences; the percentage of cells undergoing DNA synthesis and mitosis greatly decreased from 23 to 13% for S phase and from 25 to 19% for G2/M phase. This coincided with an increase in the proportion of G1 cells by about 15%. The dramatically decreased ratios observed in the mutant as compared with their parental wild types suggest that cell division is delayed due to decreased BR activities in this mutant.

### Genes of BR biosynthesis and cell division show differential expression patterns

**Figure 6:**
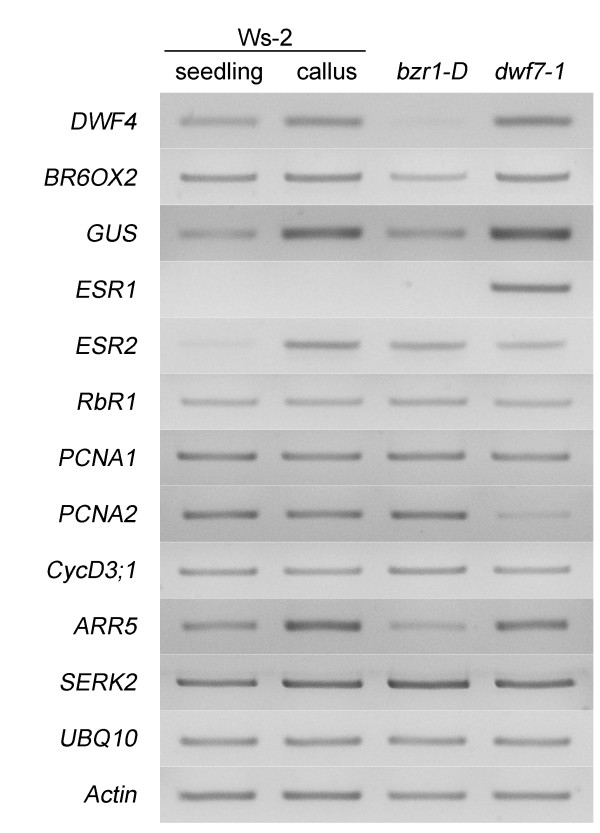
**Semi-quantitative RT-PCR analysis of the genes involved in BR biosynthesis and cell division**. Total RNA isolated from whole seedlings or calli of wild type and *dwf7-1 *were subjected to a semi-quantitative RT-PCR assay. The tested genes are listed at left side. The number of thermo cycles for each gene ranged from 24-28. Both the UBQ10 and ACTIN2 genes were used as a loading control.

To understand the differences in cell division at the molecular level, we examined the steady state levels of transcripts for the genes involved in BR biosynthesis and cell division. As a control, we included RNA from whole plants grown for 7 days in the light. Figure 6 shows the results. The expression levels of the two BR biosynthetic genes, *DWF4 *and *BR6OX2*, increased in the calli of both *dwf7-1 *and wild type (Figure 6). This demonstrates that the BR biosynthetic activity is generally upregulated in the callus stage. In contrast, the steady state levels of *RbR1*, *PCNA1*, and *Cyclin D3;1 *(*CycD3;1*) in the two calli stayed unchanged relative to the seedling control.

Previously, it was shown that two genes, *ESR2 *and *ARABIDOPSIS RESPONSE REGULATOR5 *(*ARR5*), are regulated in opposite ways during shoot induction; *ESR2 *increases but *ARR5 *decreases [50,51]. We found that *ESR2 *levels noticeably decreased in *dwf7-1 *relative to whole seedlings and wild type, whereas *ARR5 *level stayed high in *dwf7-1*. Furthermore, a gene that is putatively involved in somatic embryogenesis, *SOMATIC EMBRYOGENESIS RELATED KINASE 2 *(*SERK2*), slightly increased in the wild-type callus. In contrast, the level of *PCNA2 *transcripts representing the status of an active replication of nuclei DNA was significantly low in *dwf7-1*. The lower levels of both *PCNA2 *and *ESR2 *observed in *dwf7-1 *imply that BRs are required to induce both the two genes, and this might be associated with the lower rate of cell division and shoot induction in the *dwf7-1 *mutant, as shown in Figure 4.

## Conclusions

We have shown that *DWF4 *is strongly expressed in dividing callus cells (Figure 2). Previously, we reported that *DWF4 *was rarely expressed in the limited tissues of intact plants [49] and that its expression led to increased levels of bioactive BRs. The expression of *DWF4 *in calli suggests that BRs play important roles in supporting the dividing callus cells. In addition, we found that the portion for the number of dividing cells was smaller in the roots of the BR mutant relative to wild type, which clearly indicates that BRs are positive regulators of cell division. Furthermore, we showed that shoot induction rates, which depend on the coordination of cell differentiation processes, were lower in the BR deficient mutant, *dwf7-1*. Because the expression levels of the *ESR2 *gene were also lower in *dwf7-1 *mutant, it is likely that BRs take part in controlling shoot induction via regulation of the *ESR2 *gene. Taken all together, it is evident that BRs control both cell division and differentiation. Future research should work toward developing a detailed mechanism of BR control over sets of genes that are involved in cell division.

## Methods

### Plant material, growth conditions, and induction of callus and shoot

Previously, we reported that the promoter-reporter construct *DWF4pro:GUS *was introduced into various BR mutants by genetic crossing [49]. Here, we used a line that is homozygous for both the reporter gene and a BR mutation: *dwf7-1*, the biosynthetic mutant. This line and wild-type (Wassilewskija-2, Ws-2) seeds were sterilized and germinated on Murashige and Skoog (MS) media supplemented with 3% sucrose (pH 5.8). Plants were grown at 22°C under long-day conditions (16 h day and 8 h night).

To induce calli from each genotype, root explants (5 - 10 mm long) were placed on a callus induction medium (CIM) composed of MS salts, 0.5 mg/L 2,4-dichlorophenoxyacetic acid (2,4-D), and 0.05 mg/L Kinetin. Calli were induced at 22°C under continuous light.

Shoots were induced by growing the calli on media in 16 different combinations of auxin and cytokinin concentrations. Four different concentrations of auxin (IAA) were used, including 0.05, 0.1, 0.15, and 0.25 mg L^-1^, and the cytokinin (N^6^-Δ^2^-isopentenyladenine) concentrations used included 1, 3, 5, and 7 mg L^-1^. For each medium with the different auxin and cytokinin concentrations, calli were serially sub-cultured for 6 months, transferred and incubated for 4 weeks until visible shoots formed. This shoot induction was repeated 3 times, and 3-9 calli per each genotype were tested.

The different levels of shoot induction from each genotype were quantified. We converted the degree of shoot induction into an arbitrary numerical representation. If there was visible leaf induction observed in the tested concentration, the callus was assigned the number 2. When the callus turned green but did not produce a visible shoot, then it was given the number 1. In addition, as long as there was no sign of shoot induction, it was assigned the number 0. After converting the callus morphologies to numbers, mean values were obtained. Thus, the number 2 signified that all of the calli tested at the specific combination of auxin and cytokinin concentrations produced visible shoots, whereas the number 0 represented a lack of visible shoot formation from calli.

### Flow Cytometry Analysis

Approximately 30-50 mg of calli grown on callus induction medium was mechanically homogenized using a spatula in 250 μl of nuclei extraction buffer (solution A of the High Resolution Kit for Plant DNA, Partec, Munster, Germany). Another 250 μl of the nuclei extraction buffer was added, and the calli were further homogenized. After filtration through a 30-μm nylon sieve, 1 ml of staining solution containing the dye 4,6-diamidino-2-phenylindole-2HCl (solution B of the Kit) was added. DNA profiles were examined using a PAS flow cytometer (Partec), and the acquired data were processed using the FlooMax software (Partec), according to the supplier's protocols. For each sample, a minimum of 5,000 and a maximum of 15,000 particles were examined. To determine the standard peak positions of 2C and 4C cells, cotyledons of Arabidopsis seedlings grown for 8 days under the long-day conditions (16:8, light:dark) from which the calli originated were analyzed at least three times.

For flow cytometry of the seedling roots, three roots from 16-day-old seedlings were cut and measured. This was repeated at least 5 times for each genotype. Approximately 3000 particles were examined per reading.

### GUS histochemical analysis

A histochemical analysis of GUS expression was carried out for the detection of *DWF4pro:GUS *activity. Calli grown for 2 months on CIM were immersed into GUS staining solution (0.1 M sodium phosphate, pH 7.0; 10 mM EDTA; 0.5 mM potassium ferricyanide; 0.5 mM potassium ferrocyanide; 0.1% Triton X-100) and incubated for one hour at 37°C in darkness. The samples were serially de-stained with ethanol (50%, 70%, 90%, 100%; 30 min each). Callus staining patterns were examined under a stereomicroscope and photographed using a digital camera.

### In vivo GUS assay

Each callus weighing about 35 mg was placed into a well of a 96-well plate, and a substrate solution containing 50 mM sodium phosphate (pH 7),10 mM β-mercaptoethanol, 10 mM EDTA, 0.1% [w/v] SDS, 0.1%[w/v] Triton X-100, 2% isopropanol; and 440 mg/l 4-methylumbelliferyl β-D-glucuronide was added. The plates were incubated for 16 h at 37°C in the dark. To stop the reaction, 100 μl of ice cold 0.2 M Na_2_CO_3 _was added. The fluorescence intensity was measured using a spectrophotometer (Varian) with an excitation wavelength of 360 nm and an emission wavelength of 465 nm. To determine the relative activity of the GUS enzyme, a standard curve was constructed using different concentrations of 4-MU (4-methylumbelliferyl β-D-glucuronide). The in vivo GUS activity from each genotype was obtained, and mean values were calculated to show standard error (n < 9).

### Semi-quantitative RT-PCR analysis

Approximately 80 mg of sub-cultured calli from the CIM were ground under liquid nitrogen to a fine powder before transfer to an RNA extraction buffer containing TRIzol (Takara). Total RNA was further purified with chloroform and precipitated with isopropyl alcohol. cDNA synthesis was performed using a reverse transcriptase (Fermentas) and 2 μg of total RNA. Each template RNA was normalized using the Ubiquitin10 gene as a loading control. Oligonucleotide sequences used in this analysis are listed in additional file 2. The numbers of cycles used for DNA amplification were 17 for *UBQ10*; 24 for *PCNA2*, *RbR1*, and *CycD3;1*; 25 for *DWF4 *and *BR6Ox2*; and 28 for *PCNA1*.

## Authors' contributions

**JC **performed the experiments. **JC **and **SYP **carried out the flow cytometry experiments. **BS **analyzed the results and revised the manuscript. **SC **was the principal investigator of the project; he designed and analyzed the experiments and wrote the manuscript. All authors have read and approved the final manuscript.

## Supplementary Material

Additional file 1 **GUS staining pattern after auxin washing**. Because it was proposed that auxin affects BR responses, we examined the calli after washing off the auxin that had been added to the callus induction medium (CIM).The washed calli also displayed a similar pattern (top row) as those without auxin removal (bottom row). The similar staining pattern with or without auxin washing imply that *DWF4 *transcription is required for supporting the growth of calli. Click here for file

Additional file 2 **Oligonucleotide sequences used for semi-quantitative RT-PCR analysis**. The primer sequences are shown with respective locus ID and a melting temperature used in our PCR experiments. Click here for file

## References

[B1] Bishop GJ Brassinosteroid Mutants of Crops J Plant Growth Regul 2003 22 4 325 335 10.1007/s00344-003-0064-1 14676972

[B2] Choe S Davies PJ Brassinosteroid biosynthesis and metabolism Plant Hormones: Biosynthesis, Signal transduction, Action! 2004 Dordrecht: Kluwer Academic Publishers 156 178

[B3] Chono M Honda I Zeniya H Yoneyama K Saisho D Takeda K Takatsuto S Hoshino T Watanabe Y A semidwarf phenotype of barley uzu results from a nucleotide substitution in the gene encoding a putative brassinosteroid receptor Plant Physiol 2003 133 3 1209 1219 10.1104/pp.103.026195 14551335 PMC281616

[B4] Suzuki Y Saso K Fujioka S Yoshida S Nitasaka E Nagata S Nagasawa H Takatsuto S Yamaguchi I A dwarf mutant strain of Pharbitis nil, Uzukobito (kobito), has defective brassinosteroid biosynthesis Plant J 2003 36 3 401 410 10.1046/j.1365-313X.2003.01887.x 14617096

[B5] Vert G Nemhauser J Geldner N Hong F Chory J Molecular mechanisms of steroid hormone signaling in plants Annu Rev Cell Dev Biol 2005 21 177 201 10.1146/annurev.cellbio.21.090704.151241 16212492

[B6] Fujioka S Yokota T Biosynthesis and metabolism of brassinosteroids Annu Rev Plant Biol 2003 54 137 164 10.1146/annurev.arplant.54.031902.134921 14502988

[B7] Choe S Dilkes BP Fujioka S Takatsuto S Sakurai A Feldmann KA The *DWF4 *gene of Arabidopsis encodes a cytochrome P450 that mediates multiple 22α-hydroxylation steps in brassinosteroid biosynthesis Plant Cell 1998 10 2 231 243 10.1105/tpc.10.2.231 9490746 PMC143988

[B8] Choe S Fujioka S Noguchi T Takatsuto S Yoshida S Feldmann KA Overexpression of DWARF4 in the brassinosteroid biosynthetic pathway results in increased vegetative growth and seed yield in Arabidopsis Plant J 2001 26 6 573 582 10.1046/j.1365-313x.2001.01055.x 11489171

[B9] Choe S Dilkes BP Gregory BD Ross AS Yuan H Noguchi T Fujioka S Takatsuto S Tanaka A Yoshida S The Arabidopsis *dwarf1 *mutant is defective in the conversion of 24-methylenecholesterol to campesterol in brassinosteroid biosynthesis Plant Physiol 1999 119 3 897 907 10.1104/pp.119.3.897 10069828 PMC32104

[B10] Choe S Tanaka A Noguchi T Fujioka S Takatsuto S Ross AS Tax FE Yoshida S Feldmann KA Lesions in the sterol Δ^7 ^reductase gene of Arabidopsis cause dwarfism due to a block in brassinosteroid biosynthesis Plant J 2000 21 5 431 443 10.1046/j.1365-313x.2000.00693.x 10758495

[B11] Choe S Noguchi T Fujioka S Takatsuto S Tissier CP Gregory BD Ross AS Tanaka A Yoshida S Tax FE The Arabidopsis *dwf7/ste1 *mutant is defective in the delta7 sterol C-5 desaturation step leading to brassinosteroid biosynthesis Plant Cell 1999 11 2 207 221 10.1105/tpc.11.2.207 9927639 PMC144158

[B12] Fujioka S Li J Choi YH Seto H Takatsuto S Noguchi T Watanabe T Kuriyama H Yokota T Chory J The Arabidopsis deetiolated2 mutant is blocked early in brassinosteroid biosynthesis Plant Cell 1997 9 11 1951 1962 10.1105/tpc.9.11.1951 9401120 PMC157049

[B13] Li J Biswas MG Chao A Russell DW Chory J Conservation of function between mammalian and plant steroid 5alpha-reductases Proc Natl Acad Sci USA 1997 94 8 3554 3559 10.1073/pnas.94.8.3554 9108014 PMC20477

[B14] Li J Nagpal P Vitart V McMorris TC Chory J A role for brassinosteroids in light-dependent development of Arabidopsis Science 1996 272 5260 398 401 10.1126/science.272.5260.398 8602526

[B15] Mathur J Molnar G Fujioka S Takatsuto S Sakurai A Yokota T Adam G Voigt B Nagy F Maas C Transcription of the Arabidopsis CPD gene, encoding a steroidogenic cytochrome P450, is negatively controlled by brassinosteroids Plant J 1998 14 5 593 602 10.1046/j.1365-313X.1998.00158.x 9675902

[B16] Szekeres M Nemeth K Koncz-Kalman Z Mathur J Kauschmann A Altmann T Redei GP Nagy F Schell J Koncz C Brassinosteroids rescue the deficiency of CYP90, a cytochrome P450, controlling cell elongation and de-etiolation in Arabidopsis Cell 1996 85 2 171 182 10.1016/S0092-8674(00)81094-6 8612270

[B17] Kim GT Fujioka S Kozuka T Tax FE Takatsuto S Yoshida S Tsukaya H CYP90C1 and CYP90D1 are involved in different steps in the brassinosteroid biosynthesis pathway in Arabidopsis thaliana Plant J 2005 41 5 710 721 10.1111/j.1365-313X.2004.02330.x 15703058

[B18] Kim GT Tsukaya H Uchimiya H The ROTUNDIFOLIA3 gene of Arabidopsis thaliana encodes a new member of the cytochrome P-450 family that is required for the regulated polar elongation of leaf cells Genes Dev 1998 12 15 2381 2391 10.1101/gad.12.15.2381 9694802 PMC317051

[B19] Ohnishi T Szatmari AM Watanabe B Fujita S Bancos S Koncz C Lafos M Shibata K Yokota T Sakata K C-23 hydroxylation by Arabidopsis CYP90C1 and CYP90D1 reveals a novel shortcut in brassinosteroid biosynthesis Plant Cell 2006 18 11 3275 3288 10.1105/tpc.106.045443 17138693 PMC1693957

[B20] Kim BK Fujioka S Takatsuto S Tsujimoto M Choe S Castasterone is a likely end product of brassinosteroid biosynthetic pathway in rice Biochem Biophys Res Commun 2008 374 4 614 619 10.1016/j.bbrc.2008.07.073 18656444

[B21] Bishop GJ Harrison K Jones JD The tomato Dwarf gene isolated by heterologous transposon tagging encodes the first member of a new cytochrome P450 family Plant Cell 1996 8 6 959 969 10.1105/tpc.8.6.959 8672892 PMC161151

[B22] Bishop GJ Nomura T Yokota T Harrison K Noguchi T Fujioka S Takatsuto S Jones JD Kamiya Y The tomato DWARF enzyme catalyses C-6 oxidation in brassinosteroid biosynthesis Proc Natl Acad Sci USA 1999 96 4 1761 1766 10.1073/pnas.96.4.1761 9990098 PMC15587

[B23] Castle J Szekeres M Jenkins G Bishop GJ Unique and overlapping expression patterns of Arabidopsis CYP85 genes involved in brassinosteroid C-6 oxidation Plant Mol Biol 2005 57 1 129 140 10.1007/s11103-004-6851-7 15821873

[B24] Nomura T Kushiro T Yokota T Kamiya Y Bishop GJ Yamaguchi S The last reaction producing brassinolide is catalyzed by cytochrome P-450s, CYP85A3 in tomato and CYP85A2 in Arabidopsis J Biol Chem 2005 280 18 17873 17879 10.1074/jbc.M414592200 15710611

[B25] Mori M Nomura T Ooka H Ishizaka M Yokota T Sugimoto K Okabe K Kajiwara H Satoh K Yamamoto K Isolation and characterization of a rice dwarf mutant with a defect in brassinosteroid biosynthesis Plant Physiol 2002 130 3 1152 1161 10.1104/pp.007179 12427982 PMC166636

[B26] Shimada Y Fujioka S Miyauchi N Kushiro M Takatsuto S Nomura T Yokota T Kamiya Y Bishop GJ Yoshida S Brassinosteroid-6-oxidases from Arabidopsis and tomato catalyze multiple C-6 oxidations in brassinosteroid biosynthesis Plant Physiol 2001 126 2 770 779 10.1104/pp.126.2.770 11402205 PMC111167

[B27] Shimada Y Goda H Nakamura A Takatsuto S Fujioka S Yoshida S Organ-specific expression of brassinosteroid-biosynthetic genes and distribution of endogenous brassinosteroids in Arabidopsis Plant Physiol 2003 131 1 287 297 10.1104/pp.013029 12529536 PMC166808

[B28] Tanaka K Asami T Yoshida S Nakamura Y Matsuo T Okamoto S Brassinosteroid homeostasis in Arabidopsis is ensured by feedback expressions of multiple genes involved in its metabolism Plant Physiol 2005 138 2 1117 1125 10.1104/pp.104.058040 15908602 PMC1150425

[B29] Clouse SD Langford M McMorris TC A brassinosteroid-insensitive mutant in Arabidopsis thaliana exhibits multiple defects in growth and development Plant Physiol 1996 111 3 671 678 10.1104/pp.111.3.671 8754677 PMC157882

[B30] Li J Chory J A putative leucine-rich repeat receptor kinase involved in brassinosteroid signal transduction Cell 1997 90 5 929 938 10.1016/S0092-8674(00)80357-8 9298904

[B31] Wang ZY Seto H Fujioka S Yoshida S Chory J BRI1 is a critical component of a plasma-membrane receptor for plant steroids Nature 2001 410 6826 380 383 10.1038/35066597 11268216

[B32] Kinoshita T Cano-Delgado A Seto H Hiranuma S Fujioka S Yoshida S Chory J Binding of brassinosteroids to the extracellular domain of plant receptor kinase BRI1 Nature 2005 433 7022 167 171 10.1038/nature03227 15650741

[B33] Wang X Chory J Brassinosteroids regulate dissociation of BKI1, a negative regulator of BRI1 signaling, from the plasma membrane Science 2006 313 5790 1118 1122 10.1126/science.1127593 16857903

[B34] Kim TW Guan S Sun Y Deng Z Tang W Shang JX Burlingame AL Wang ZY Brassinosteroid signal transduction from cell-surface receptor kinases to nuclear transcription factors Nat Cell Biol 2009 11 10 1254 1260 10.1038/ncb1970 19734888 PMC2910619

[B35] Choe S Schmitz RJ Fujioka S Takatsuto S Lee MO Yoshida S Feldmann KA Tax FE Arabidopsis brassinosteroid-insensitive *dwarf12 *mutants are semidominant and defective in a glycogen synthase kinase 3β-like kinase Plant Physiol 2002 130 3 1506 1515 10.1104/pp.010496 12428015 PMC166669

[B36] Li J Nam KH Regulation of brassinosteroid signaling by a GSK3/SHAGGY-like kinase Science 2002 295 5558 1299 1301 11847343 10.1126/science.1065769

[B37] Peng P Yan Z Zhu Y Li J Regulation of the Arabidopsis GSK3-like Kinase BRASSINOSTEROID-INSENSITIVE 2 through Proteasome-Mediated Protein Degradation Mol Plant 2008 1 2 338 346 10.1093/mp/ssn001 18726001 PMC2519614

[B38] Perez-Perez JM Ponce MR Micol JL The ULTRACURVATA2 gene of Arabidopsis encodes an FK506-binding protein involved in auxin and brassinosteroid signaling Plant Physiol 2004 134 1 101 117 10.1104/pp.103.032524 14730066 PMC316291

[B39] Wang ZY Nakano T Gendron J He J Chen M Vafeados D Yang Y Fujioka S Yoshida S Asami T Nuclear-localized BZR1 mediates brassinosteroid-induced growth and feedback suppression of brassinosteroid biosynthesis Dev Cell 2002 2 4 505 513 10.1016/S1534-5807(02)00153-3 11970900

[B40] Yin Y Vafeados D Tao Y Yoshida S Asami T Chory J A new class of transcription factors mediates brassinosteroid-regulated gene expression in Arabidopsis Cell 2005 120 2 249 259 10.1016/j.cell.2004.11.044 15680330

[B41] Yin Y Wang ZY Mora-Garcia S Li J Yoshida S Asami T Chory J BES1 accumulates in the nucleus in response to brassinosteroids to regulate gene expression and promote stem elongation Cell 2002 109 2 181 191 10.1016/S0092-8674(02)00721-3 12007405

[B42] He JX Gendron JM Sun Y Gampala SS Gendron N Sun CQ Wang ZY BZR1 is a transcriptional repressor with dual roles in brassinosteroid homeostasis and growth responses Science 2005 307 5715 1634 1638 10.1126/science.1107580 15681342 PMC2925132

[B43] Mandava NB Plant Growth-Promoting Brassinosteroids Ann Rev Plant Physiol 1988 39 23 52 10.1146/annurev.pp.39.060188.000323

[B44] Willemse J Kulikova O de Jong H Bisseling T A new whole-mount DNA quantification method and the analysis of nuclear DNA content in the stem-cell niche of Arabidopsis roots Plant J 2008 55 5 886 894 10.1111/j.1365-313X.2008.03548.x 18466307

[B45] Francis D The plant cell cycle--15 years on New Phytol 2007 174 2 261 278 10.1111/j.1469-8137.2007.02038.x 17388890

[B46] Yoo MJ Albert VA Soltis PS Soltis DE Phylogenetic diversification of glycogen synthase kinase 3/SHAGGY-like kinase genes in plants BMC Plant Biol 2006 6 3 10.1186/1471-2229-6-3 16504046 PMC1524769

[B47] Hu Y Bao F Li J Promotive effect of brassinosteroids on cell division involves a distinct CycD3-induction pathway in Arabidopsis Plant J 2000 24 5 693 701 10.1046/j.1365-313x.2000.00915.x 11123807

[B48] Ibanes M Fabregas N Chory J Cano-Delgado AI Brassinosteroid signaling and auxin transport are required to establish the periodic pattern of Arabidopsis shoot vascular bundles Proc Natl Acad Sci USA 2009 106 32 13630 13635 10.1073/pnas.0906416106 19666540 PMC2717112

[B49] Kim HB Kwon M Ryu H Fujioka S Takatsuto S Yoshida S An CS Lee I Hwang I Choe S The regulation of *DWARF4 *expression is likely a critical mechanism in maintaining the homeostasis of bioactive brassinosteroids in Arabidopsis Plant Physiol 2006 140 2 548 557 10.1104/pp.105.067918 16407451 PMC1361323

[B50] Banno H Ikeda Y Niu QW Chua NH Overexpression of Arabidopsis ESR1 induces initiation of shoot regeneration Plant Cell 2001 13 12 2609 2618 10.1105/tpc.13.12.2609 11752375 PMC139476

[B51] Ikeda Y Banno H Niu QW Howell SH Chua NH The ENHANCER OF SHOOT REGENERATION 2 gene in Arabidopsis regulates CUP-SHAPED COTYLEDON 1 at the transcriptional level and controls cotyledon development Plant Cell Physiol 2006 47 11 1443 1456 10.1093/pcp/pcl023 17056621

