# Supplementary material for: Arabidopsis brassinosteroid biosynthetic mutant dwarf7-1 exhibits slower rates of cell division and shoot induction
Source: BMC Plant Biol. 2010 Dec 9;10:270. doi: 10.1186/1471-2229-10-270 (PMC3017067; doi:10.1186/1471-2229-10-270)

**Additional files**

**Additional file 1.** GUS staining pattern after auxin washing. Because it was proposed that auxin affects BR responses, we examined the calli after washing off the auxin that had been added to the callus induction medium (CIM).The washed calli also displayed a similar pattern (top row) as those without auxin removal (bottom row). The similar staining pattern with or without auxin washing imply that *DWF4* transcription is required for supporting the growth of calli.


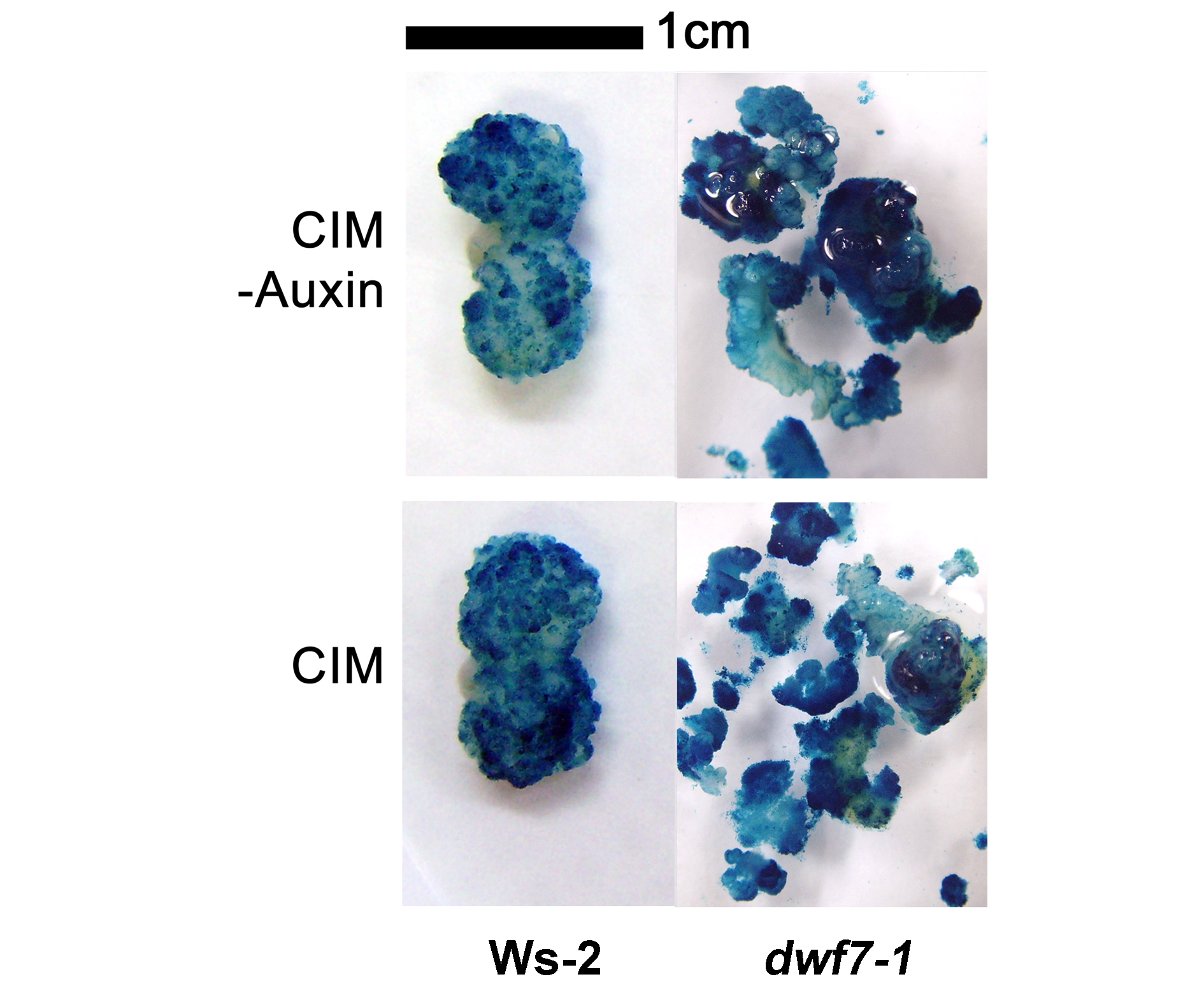

Supplement: Additional file 1 — GUS staining pattern after auxin washing. Because it was proposed that auxin affects BR responses, we examined the calli after washing off the auxin that had been added to the callus induction medium (CIM).The washed calli also displayed a similar pattern (top row) as those without auxin removal (bottom row). The similar staining pattern with or without auxin washing imply that DWF4 transcription is required for supporting the growth of calli. [file 1471-2229-10-270-S1.DOC]
