# Supplementary material for: Arabidopsis brassinosteroid biosynthetic mutant dwarf7-1 exhibits slower rates of cell division and shoot induction
Source: BMC Plant Biol. 2010 Dec 9;10:270. doi: 10.1186/1471-2229-10-270 (PMC3017067; doi:10.1186/1471-2229-10-270)
Supplement: Additional file 2 — Oligonucleotide sequences used for semi-quantitative RT-PCR analysis. The primer sequences are shown with respective locus ID and a melting temperature used in our PCR experiments. [file 1471-2229-10-270-S2.DOC]

**Additional files**

**Additional file 2.** Oligonucleotide sequences used for semi-quantitative RT-PCR analysis. The primer sequences are shown with respective locus ID and a melting temperature used in our PCR experiments.

| Gene Symbol | Locus ID | Primer Sequence | Tm (˚C) |
| --- | --- | --- | --- |
| *DWARF4* | At3G50660 | 5’-AGATGTTCGGTACAAAGGATACGATATC-3’ | 57 |
|  |  | 5’-GTTTATCATCTTCTGCTAATTCCCAATTG-3’ |  |
| *BR6Ox2* | At3G30180 | 5’-GACATCAAGATTGGCAACGA-3’ | 57 |
|  |  | 5’-TTAGCCCTACAAAATGACCCTT-3’ |  |
| *GUS* | NA | 5’-GTTCTGCGACGCTCACACCGAT-3’ | 62 |
|  |  | 5’-GCAGCCCGGCTAACGTATCCAC-3’ |  |
| *ESR1* | At1G12980 | 5’-TTCCTCTCGTAAAACGACTCCG-3’ | 62 |
|  |  | 5’-TCCCCACGATCTTCGGCAAG-3’ |  |
| *ESR2* | At1G24590 | 5’-ACTTTCGCTTACCCGCCTTGT-3’ | 60 |
|  |  | 5’-CATTCTCATGATCAGCCCAACC-3’ |  |
| *RbR1* | At3G12280 | 5’-TCTGCATTTGCCAGCCCAACA-3’ | 53 |
|  |  | 5’-ATTGACCTTCAGGCTTATTATTGGC-3’ |  |
| *PCNA1* | At1G07370 | 5’-TCATGTTTGAGAGCCCCACG-3’ | 52 |
|  |  | 5’-GTCTTCTTCTTCTTCAATCTTAGGA-3’ |  |
| *PCNA2* | At2G29570 | 5’-GAGTCCCACACAAGACAAGATTG-3’ | 52 |
|  |  | 5’-TCTTCTTCAATCTTAGGCGCCAG-3’ |  |
| *CycD3;1* | At4G34160 | 5’-TTCTAGACTTTCAAGTGGAGGAG-3’ | 53 |
|  |  | 5’-AATCGTTTGAGCTTTCGTCGCTATT-3’ |  |
| *ARR5* | At3G48100 | 5’-AGCGGTTACTCAGAGTCTCAT-3’ | 59 |
|  |  | 5’-CTTAAAAGCTCTTTCCTCAGCT-3’ |  |
| *SERK2* | At1G34210 | 5’-GTCGCTTCCTGTTTGAG-3’ | 57 |
|  |  | 5’-TCTTGGACCAGACAACTCCAT-3’ |  |
| *UBQ10* | At4G05320 | 5’-GATCTTTGCCGGAAAACAATTGGAGGATGGT-3’ | 62 |
|  |  | 5’-CATTCTCATGATCAGCCCAACC-3’ |  |
